# A Meta-Analysis of the Efficacy and Safety of the 0.19 mg Fluocinolone Acetonide Implant in Non-Infectious Uveitis

**DOI:** 10.3390/biomedicines13020248

**Published:** 2025-01-21

**Authors:** Suji Yeo, Yoo-Ri Chung, Ji Hun Song, Bahram Bodaghi, Sara Touhami

**Affiliations:** 1 Department of Ophthalmology, Ajou University School of Medicine, Suwon 16499, Republic of Korea; 2 Department of Ophthalmology, Pitié-Salpêtrière University Hospital, Sorbonne University, 75013 Paris, France; 3 Department of Ophthalmology, Tenon University Hospital, Sorbonne University, 75020 Paris, France

**Keywords:** fluocinolone acetonide, non-infectious uveitis, meta-analysis

## Abstract

**Background/Objectives:** The fluocinolone acetonide implant (FAI) is an intravitreal corticosteroid implant designed to have a therapeutic effect lasting up to 3 years. We performed a meta-analysis to investigate the efficacy and safety of the FAI (0.19 mg, releasing at 0.2 μg/day) in patients with non-infectious uveitis. **Methods**: The PubMed, EMBASE, and Cochrane Library databases were last searched on 6 September 2024. Studies comparing FAI with sham injections were investigated. The primary outcome was the recurrence of uveitis. Secondary outcomes included visual acuity, intraocular pressure (IOP), and occurrence of cataracts. **Results**: Significantly more patients in the FAI group experienced no uveitis recurrence for up to 36 months compared to the sham group, with a relatively lower number of recurrences. Systemic adjuvant therapy was similar between groups, while fewer patients required local rescue injections in the FAI group (95% confidence interval (CI): −2.91 to −1.70). Visual acuity changes and the proportion of eyes with ≥15 letters gain were not significantly different between the groups. More patients needed cataract surgery in the FAI group (95% CI: 0.68–1.96). No differences were observed in IOP change, final IOP, or treatment-requiring events related to an increased IOP. However, more subjects experienced events of IOP > 25 mmHg with the FAI (95% CI: 0.73 to 2.14). **Conclusions**: The 0.19 mg FAI was effective in preventing uveitis recurrence, and reduced the need for local injections. No significant impacts were noted in terms of systemic therapy, visual improvement, or most IOP-related complications.

## 1. Introduction

The fluocinolone acetonide implant (FAI) is designed to release submicrogram doses of corticosteroids into the vitreous cavity lasting up to 3 years [1,2]. This allows a theoretically smaller number of intravitreal injections, which can reduce the treatment burden for both patients and clinicians. The FAI is approved for the treatment of chronic diabetic macular edema (DME) that does not respond sufficiently to available therapies and for the prevention of relapse in chronic non-infectious uveitis (NIU) [1,2]. In NIU, current therapeutic strategies focus on the control of active inflammation and the prevention of recurrences [3]. Traditional therapeutic options include systemic corticosteroids and/or immunomodulatory agents, which can both induce systemic side effects. On the other hand, local treatments such as dexamethasone implants offer limited long-term efficacy, lasting up to six months [1]. Fluocinolone acetonide implants were therefore developed to provide a long-lasting local anti-inflammatory effect while avoiding systemic adverse events. Avoiding cumulative damage caused by recurrent episodes of inflammation is crucial to reduce irreversible damage and long-term complications [4].

The FAI that releases at 0.2 μg/day includes two implants: one containing 0.19 mg (Iluvien^®^; Alimera Sciences, Dublin, Ireland) and the other containing 0.18 mg (Yutiq^®^; Alimera Sciences Inc., Alpharetta, GA, USA) [3,5]. Both implants allow a steady-state concentration of 0.5 to 1.0 ng/mL to be reached within 6 to 9 months [1]. In contrast, the FAI containing 0.59 mg (Retisert^®^; Bausch and Lomb, Rochester, NY, USA) is designed to release at 0.6 μg/day over the first month, and then release a decreased dose of 0.03–0.04 μg/day over approximately 30 months [6]. The 0.19 mg FAI (Iluvien^®^) considered in this study is a nonbiodegradable cylindrical implant measuring 3.5 mm in length and 0.37 mm in diameter. It consists of a polyvinyl alcohol permeable membrane and an internal matrix containing fluocinolone acetonide [7]. The implant can be inserted into the vitreous cavity through the inbuilt 25-gauge needle of the device.

Besides the well-known risks of cataracts and glaucoma, there are other complications that can arise with FAIs. These include ocular hypotony, choroidal detachment, vitreous hemorrhage, retinal detachment, and implant migration to the anterior chamber [8,9], as previously reported with dexamethasone implants. The risk of implant migration is higher in eyes with previous vitrectomy, an open/defective lens capsule, and/or iris defects [10]. Persistent floaters can also cause discomfort as the implant remains permanently within the vitreous cavity. Another limitation of the use of FAI is its high cost. Although it was suggested to be cost-effective in NIU patients with unilateral disease, as it reduced the number of recurrences [11,12], this was not the case in patients with bilateral involvement [13].

A meta-analysis that compared various intravitreal agents for NIU reported that no specific regimen showed a significant superiority, whereas the 0.59 mg FAI was associated with a higher risk of cataract and intraocular pressure (IOP) rise [2]. All FAIs (including the 0.19 mg and 0.59 mg dosages) were associated with a lower risk of uveitis recurrence, while no significant differences were noted in terms of visual improvement compared to standard care [2]. Various real-world studies investigating the efficacy and safety of FAI in NIU-related macular edema (ME) noted significant anatomical improvements, but controversial results were reported regarding visual acuity outcomes [14,15,16,17,18,19,20].

Relatively fewer controlled studies and meta-analyses focus on NIU compared to DME [5,21]. Currently, two randomized controlled studies (RCTs) have investigated the efficacy and safety of FAI (0.2 μg/day) in NIU for up to 36 months [22,23]. They both reported significantly less uveitis recurrence and less use of local adjuvant injections, and no differences in the use of systemic adjuvant therapy or IOP-related events were noted [22,23]. Meanwhile, despite their similar design, discrepancies exist regarding the number of uveitis recurrences and outcomes associated with visual improvement [22,23]. Despite the limited number of available studies, we considered it meaningful to conduct a meta-analysis as the two RCTs showed different results in factors related to visual improvement. Accordingly, we investigated the efficacy and safety of FAI (0.19 mg) in patients with NIU through a literature search and meta-analysis.

## 2. Materials and Methods

### 2.1. Search Methods

**Figure 1:**
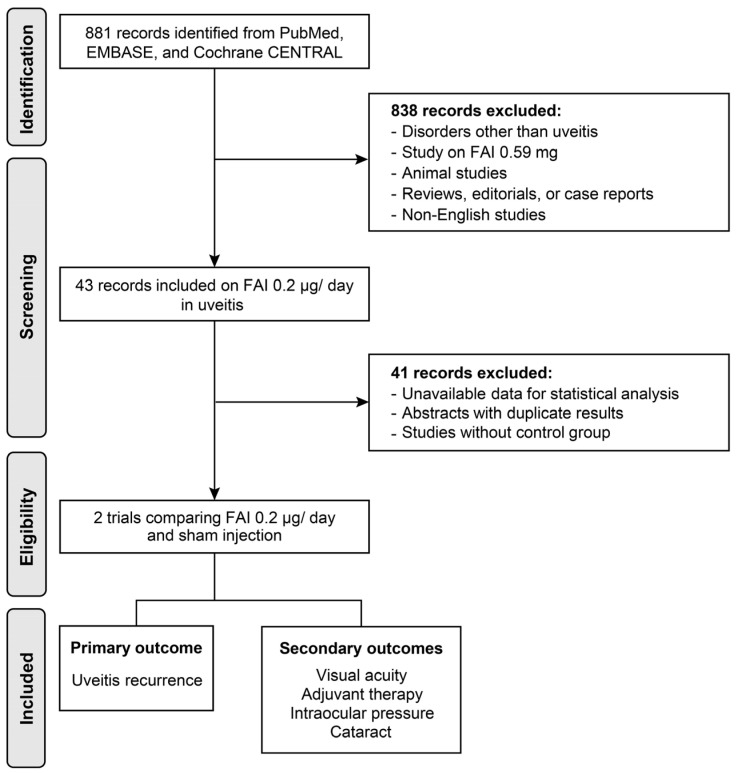
Flow diagram of the study selection process. FAI: fluocinolone acetonide implant.

The PubMed, EMBASE, and Cochrane Library databases were last searched on 6 September 2024, using the following terms as principal keywords: ‘uveitis’, ‘inflammation’, ‘fluocinolone acetonide’, ‘intravitreal injection’, ‘implant’, ‘corticosteroid’, ‘steroid’, and ‘macular edema’ (see Supplementary File S1 for details). A total of 881 studies were identified in the preliminary search. Studies comparing FAI and sham injections were investigated for meta-analysis, and further exclusion was performed based on the following criteria: (1) studies involving disorders other than uveitis; (2) studies on 0.59 mg of FAI or other corticosteroid agents; (3) animal studies, case reports, or review articles; (4) duplicated articles (including congress abstracts that were lately published in articles); and (5) studies written in non-English languages (Figure 1). This review is registered in the International Prospective Register of Systematic Reviews (PROSPERO) under registration number CRD42025634703.

### 2.2. Assessment of Potential Bias

The risk of bias in included studies was assessed on 2 December 2024 using the study quality assessment tool developed by the National Heart, Lung, and Blood Institute (NHLBI, https://www.nhlbi.nih.gov/health-topics/study-quality-assessment-tools). Each criterion was assessed by two independent reviewers (Y.R.C. and S.T.) and then discussed for overall quality rate depending on the proportion of fulfilled criteria (poor: <50%, fair: 50–75%, and good: ≥75%).

### 2.3. Primary and Secondary Outcomes

The primary outcome was the recurrence of uveitis. The secondary outcomes were visual acuity measured by the Early Treatment Diabetic Retinopathy Study (ETDRS) letters, any adjuvant therapy, and complications including cataracts or increased IOP. Systemic adjuvant therapy included corticosteroid or immunosuppressive therapy, while local adjuvant therapy included intraocular or periocular corticosteroid injections.

### 2.4. Statistical Analysis

Data on the proportions, mean values, and standard deviations were obtained from the literature. The meta-analysis was conducted using RevMan 5.4, and heterogeneity was examined using *I*^2^ statistics. The fixed model was applied as few studies were included in this meta-analysis [24]. Squares indicate the mean difference estimates for continuous variables and odds ratio (OR) for categorical variables, and lines extending from the squares represent the associated 95% confidence intervals (CI) in the forest plots. CIs that do not intersect the vertical line at 0 for continuous variables and at 1 for categorical variables indicated statistical significance at *p* < 0.05.

## 3. Results

### 3.1. Results of the Literature Search

Among the records that investigated the FAI (0.19 mg) in uveitis, two RCTs comparing FAI and sham injections were identified and used for the meta-analysis [22,23]. The characteristics and brief results of these RCTs and other excluded studies without a control group are summarized in Table 1. Briefly, most of the included studies of retrospective non-comparative design revealed the effective control of inflammation and manageable IOP during the post-implantation period. In terms of visual acuity, some of these studies reported stable vision [25,26], whereas other case series reported visual improvement with FAI. 

### 3.2. Quality Assessment

The risk of bias, assessed using the NHLBI tool, is presented in Supplementary Table S1. Overall, the selected RCTs demonstrated good quality for inclusion in the meta-analysis.

### 3.3. Outcomes

**Figure 2:**
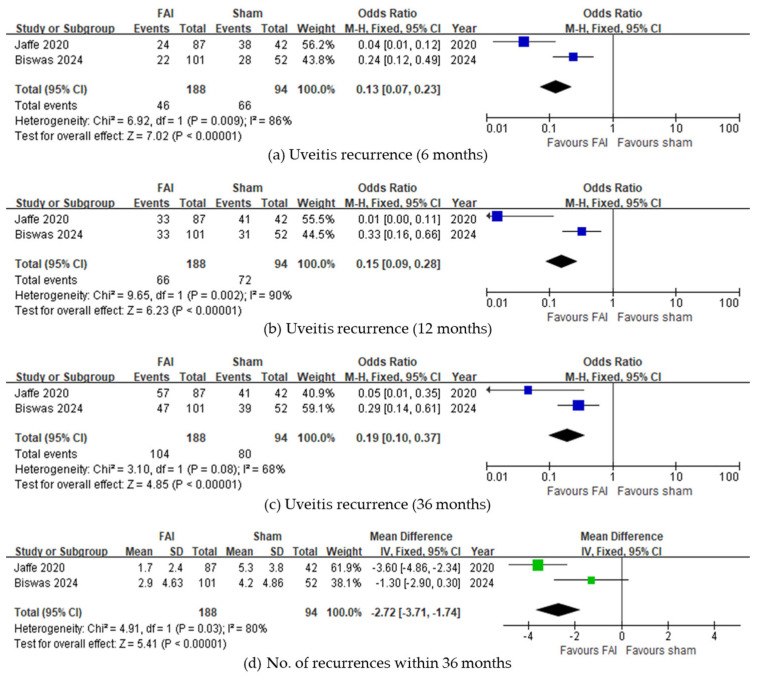
Forest plots of the odds ratios and mean difference in uveitis recurrence-related outcomes. Proportion of subjects with uveitis recurrence for (**a**) 6, (**b**) 12, and (**c**) 36 months. (**d**) Number of uveitis recurrences within 36 months. The studies labeled as “Jaffe 2020” and “Biswas 2024” correspond to Ref. [22] and Ref. [23], respectively. The blue squares represent the effect estimate for dichotomous data, while the green squares represent the effect estimate for continuous data. CI: confidence interval; FAI: fluocinolone acetonide implant; SD: standard deviation.

Regarding uveitis recurrence, fewer subjects experienced recurrence with the FAI compared to sham injections up to 6 months (95% CI 0.07–0.23, *p* < 0.001, Figure 2a), 12 months (95% CI 0.09–0.28, *p* < 0.001, Figure 2b), and 36 months (95% CI 0.10–0.37, *p* < 0.001, Figure 2c). Overall, fewer recurrences were observed in subjects with the FAI (95% CI −3.71 to −1.74, *p* < 0.001, Figure 2d). Regarding ME, both studies reported fewer eyes with ME at 36 months, although the meta-analysis was not conducted due to a lack of data.

**Figure 3:**
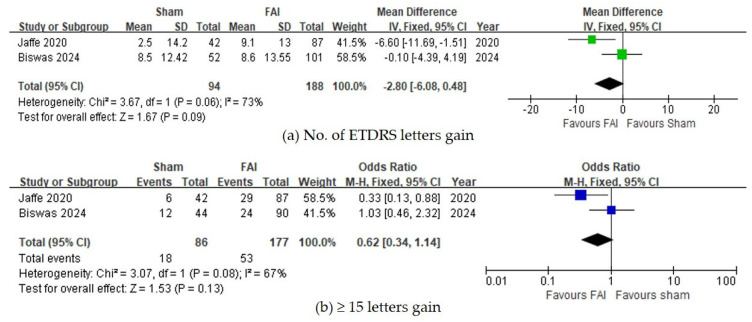
Forest plots of the mean differences in visual acuity. (**a**) Change of best-corrected visual acuity by ETDRS letters over a 36-month period. (**b**) Proportion of subjects with ≥15 ETDRS letter gains over a 36-month period. The studies labeled as “Jaffe 2020” and “Biswas 2024” correspond to Ref. [22] and Ref. [23], respectively. The blue squares represent the effect estimate for dichotomous data, while the green squares represent the effect estimate for continuous data. CI: confidence interval; ETDRS: Early Treatment Diabetic Retinopathy Study; FAI: fluocinolone acetonide implant; SD: standard deviation.

For visual acuity, no significant differences were observed between the FAI and sham for the number of gained ETDRS letters (95% CI −0.48 to 6.08, *p* = 0.09, Figure 3a) and for the proportion of subjects gaining ≥15 ETDRS letters (95% CI 0.87–2.95, *p* = 0.13, Figure 3b).

**Figure 4:**
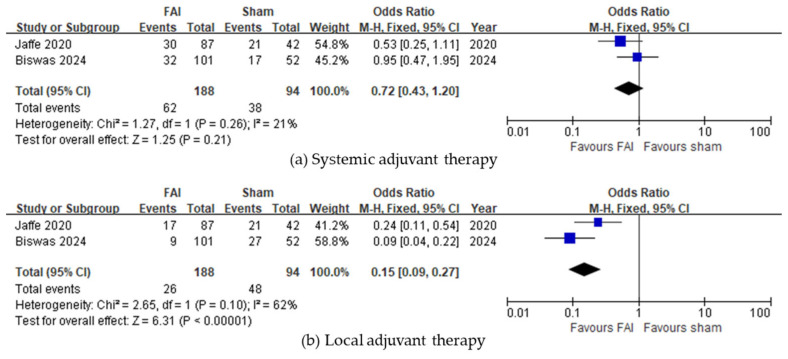
Forest plots of the mean differences in adjuvant therapy. Proportion of subjects receiving adjuvant therapy in the form of (**a**) systemic treatment or (**b**) local injections over a 36-month period. The studies labeled as “Jaffe 2020” and “Biswas 2024” correspond to Ref. [22] and Ref. [23], respectively. The blue squares represent the effect estimate for dichotomous data. CI: confidence interval; FAI: fluocinolone acetonide implant.

Regarding the need for any adjuvant therapy, the FAI was associated with a reduced need for local injections (95% CI 0.09–0.27, *p* < 0.001, Figure 4b), whereas no difference was observed regarding systemic treatment (95% CI 0.43–1.20, *p* = 0.21, Figure 4a).

**Figure 5:**
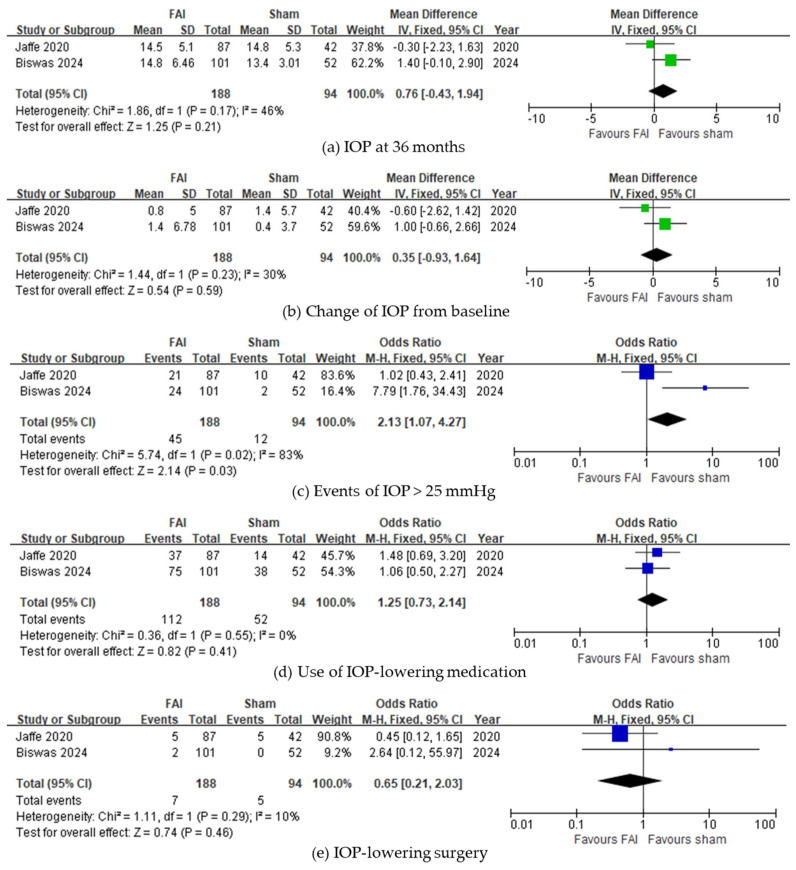
Forest plots of the mean differences in IOP-related events. (**a**) IOP at month 36 (mmHg). (**b**) Change of IOP from baseline over a 36-month period. (**c**–**e**) Proportion of subjects (**c**) with IOP events of >25 mmHg, (**d**) requiring IOP-lowering medication, and (**e**) requiring IOP-lowering surgery up to month 36. The studies labeled as “Jaffe 2020” and “Biswas 2024” correspond to Ref. [22] and Ref. [23], respectively. The blue squares represent the effect estimate for dichotomous data, while the green squares represent the effect estimate for continuous data. CI: confidence interval; FAI: fluocinolone acetonide implant; IOP: intraocular pressure; SD: standard deviation.

In IOP-related events, subjects with the FAI experienced more events of IOP > 25 mmHg (95% CI 0.73–2.14, *p* = 0.03, Figure 5c). However, no differences were noted in the final IOP at 36 months (95% CI −0.43 to 1.94, *p* = 0.21, Figure 5a) and change of IOP from baseline (95% CI −0.93 to 1.64, *p* = 0.59, Figure 5b). The use of IOP-lowering treatments was not significantly different between subjects treated with the FAI and those treated with sham injections (IOP-lowering medications: 95% CI 0.73–2.14, *p* = 0.41, Figure 5d; IOP-lowering surgery: 95% CI 0.21–2.03, *p* = 0.46, Figure 5e).

**Figure 6:**
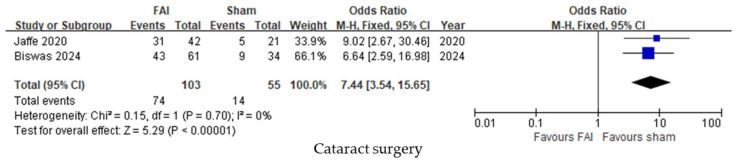
Forest plots of the mean differences in cataract surgery up to month 36. The studies labeled as “Jaffe 2020” and “Biswas 2024” correspond to Ref. [22] and Ref. [23], respectively. The blue squares represent the effect estimate for dichotomous data. CI: confidence interval; FAI: fluocinolone acetonide implant.

Cataract surgery was performed more frequently in subjects treated with the FAI compared to sham injections (95% CI 3.54–15.65, *p* < 0.001, Figure 6).

## 4. Discussion

This study conducted a meta-analysis on the efficacy and safety of the 0.19 mg FAI (releasing at 0.2 μg/day) using two RCTs of similar design [22,23]. In terms of NIU recurrence, the FAI was obviously beneficial for the prevention of NIU recurrence, both in the proportion of subjects that experienced recurrence and in the mean number of recurrences up to 3 years. The efficacy of the FAI in preventing NIU recurrence was evident and has also been reported in non-comparative real-world studies [26,27], including those reported in this paper. The reduced recurrence was also noted in studies involving specific ocular inflammatory disorders, such as Vogt–Koyanagi–Harada disease [25].

Regarding visual improvement, some discrepancies existed between both RCTs, as one study reported the benefits of FAIs whereas the other study did not. This meta-analysis revealed that no significant benefits were noted with the FAI in terms of visual gain, both in the final visual gain by ETDRS letters and the proportion of patients gaining ≥15 letters. This might be due to the different proportion of eyes undergoing cataract surgery between the included RCTs, as it significantly improves the long-term visual acuity in eyes with ocular inflammatory diseases [35]. It is also to note that the study by Biswas et al. [23], which reported no differences in visual outcomes between the FAI and sham groups, included patients with a shorter duration of uveitis compared to those involved in the study by Jaffe et al. [22] (approximately 3.3 vs. 7.1 years). Visual impairment is expected to be more frequent in patients with NIU and longer disease duration as irreversible damage is more likely to occur, especially in NIU cases affecting the posterior segment with macular involvement [36]. Another potential explanation for the absence of a difference in visual improvement between the FAI and sham groups could be the definition of visual outcomes. The two RCTs included in this meta-analysis evaluated the proportion of patients achieving a gain of ≥15 letters over a period of up to three years, whereas most retrospective case series reported smaller degrees of visual improvement [16,18,34]. Moreover, most real-world non-comparative studies reported visual improvement for up to 2 years [14,16,17]; thus, further investigations are needed to confirm the long-term efficacy of FAI in terms of visual outcomes.

Studies evaluating the control of inflammation with detailed scores reported that anterior chamber cells and vitreous haze were improved with the FAI [31,34]. One study that investigated the efficacy of FAI in birdshot retinochoroidopathy also revealed that vascular leakage was improved on fluorescein angiography along with functional improvements on electroretinograms [29]. Another indicator of effective inflammation control is the need for any adjuvant therapy. This meta-analysis showed that local treatment was less needed in FAI-treated patients, whereas no change was noted in the use of systemic adjuvant therapy. This suggests that the FAI is beneficial in lightening the burden of treatment for clinicians and patients by reducing the need for local injections [30]. Some real-world studies reported that systemic adjunctive therapy could be decreased or even discontinued in FAI-treated eyes [17,31]. We should, however, consider the relationship between NIU and various systemic autoimmune disorders that may still require systemic treatments.

The efficacy of the FAI on ME was not investigated in this meta-analysis due to the unsuitability of reported results in the included RCTs. Overall, only a few eyes with ME were treated with the FAI in both trials. However, various real-world studies reported an improved central macular thickness with stable/improved vision [20]. Cai et al. [28] reported that NIU-related ME recurred in 16% of cases treated with FAI, with a mean time-to-recurrence of 37 months. In the study involving birdshot retinochoroiditis, FAI was able to resolve ME in all patients by 6 months [19]. Comparative studies are therefore needed to clarify the exact efficacy of the FAI in NIU patients with ME.

Among the non-controlled small-sized studies published in the literature, one investigated the differences between responders and non-responders to the FAI, suggesting that older age, higher need for previous injections of dexamethasone implant, and defects in the external limiting membrane and ellipsoid zone were more frequent in non-responders [33]. The present meta-analysis could not investigate any predictive factors of FAI efficacy. Thus, further studies are needed to identify patients that would benefit most from the implant.

In terms of safety, most variables associated with IOP were not significantly different between the 0.19 mg FAI and sham injections. One study comparing the IOP change with the dexamethasone implant and subsequent FAI reported that a significant increase was noted with dexamethasone, but not with FAI [32]. Most real-world studies also reported a non-significant IOP change or change manageable with standard therapy after the FAI [19,20,26,34]. Unlike the FAI using a higher dose (0.59 mg) that needs more glaucoma surgery than dexamethasone implants [37], IOP changes seemed to be controllable with the FAI containing 0.19 mg.

Cataract, which is a well-known complication of corticosteroids, was inevitable with the use of FAI [25,30]. Although cataract is a surgically treatable ocular condition, uveitic eyes are more prone to experience postoperative complications such as ME and epiretinal membranes [38,39]. Therefore, given this higher risk of postoperative complications [38,39], the decision to inject FAI must be made carefully and is usually not the first choice in young phakic patients.

The main limitations of this meta-analysis are the small number of included studies and its restriction to publications written in English. Although we included two RCTs with similar designs, some discrepancies were noted with regard to the duration of uveitis, patients’ age at enrollment, and the proportion of patients under systemic treatment at baseline. In the present work, analysis was limited to three time points (months 6, 12, and 36) due to the design of the included studies. More frequent follow-up intervals might reveal different outcomes regarding uveitis recurrences, which represents a potential bias that should also be acknowledged. The strength of this study lies in its comprehensive analysis of outcomes with reported discrepancies. Since most studies in the literature consist of non-controlled case series, we aimed to discuss these case series alongside the interpretations derived from the current meta-analysis.

## 5. Conclusions

In conclusion, the 0.19 mg FAI (0.2 μg/day) was effective in preventing NIU recurrence; however, its impact on visual acuity remained unclear, as no significant differences were observed between the FAI and the sham treatment. Additionally, the FAI did not appear to reduce the need for adjuvant systemic therapy, as systemic treatment usage was similar across groups. Nonetheless, IOP-related events could be controlled without significant surgical intervention, though cataract progression was observed in phakic eyes.

## Figures and Tables

**Table 1:** Summary of studies investigating the efficacy and safety of FAI.

Authors	Year, Place, Study Type	Study Period	Study Group and Included Eyes	Results	Conclusion of the Study
Jaffe et al. [27]	2016, USA, Prospective, non-comparative study	24 M	- NIU (intermediate, posterior, or panuveitis) - 11 eyes (11 patients)	- VA: improved +0.25 logMAR at 12 M, +0.17 logMAR at 24 M - CMT: reduction of 26% at 12 M, 29% at 24 M - No recurrence of inflammation - No additional local injection - IOP-lowering drops needed in 18%	- Effective control of inflammation - Improved VA - Manageable IOP by standard therapy
Heo et al. [25]	2016, USA, Post hoc subgroup analysis of two RCTs	36 M	- Bilateral VKH - 60 eyes (30 patients): FAI in study eye vs. non-FAI in fellow eye	- Similar VA in both groups - Reduced recurrence in FAI eyes vs. non-FAI fellow eyes (33% vs. 87%) - Reduced dose of systemic corticosteroids after FAI than before FAI - Frequent IOP rises in FAI eyes (70% vs. 20%) - Cataracts in all phakic FAI eyes	- Effective control of inflammation - Less systemic therapy - Frequent cataract and IOP elevation
Weber et al. [20]	2019, Germany, Retrospective, non-comparative study	Mean 19 M (8–42 M)	- NIU-ME - 11 eyes (8 patients)	- VA: improved in 9/11 eyes - CMT: improved by 168 ± 202 μm - Inactive inflammation in 9/11 eyes - IOP: increase of 2.1 ± 4.7 mmHg - Cataract surgery in 2/2 eyes	- Effective control of inflammation - Improved VA and CMT - Manageable IOP and cataract
Jaffe et al. [22]	2020, USA, Randomized controlled trial	36 M	- NIU - 129 eyes (129 patients): FAI (87 eyes) vs. sham (42 eyes)	- VA improved in FAI eyes - Less recurrence at 6 M, 12 M, and 36 M in FAI eyes - Less local adjuvant therapy in FAI eyes - No significant difference in IOP-related variables - More cataracts in FAI eyes	- Effective control of inflammation - Improved VA, CMT - Manageable IOP
Cai et al. [28]	2020, USA, Retrospective, non-comparative study	Mean 34 M (12–56 M)	- NIU (intermediate, posterior, or panuveitis) - 12 eyes (12 patients)	- NIU recurrence in 42% (time to recur: mean 36 M) - ME occurrence in 16% (time to occur: mean 37 M)	- Effective control of inflammation
Hikal et al. [19]	2021, Germany, Retrospective, non-comparative study	Mean 18 M (1–60 M)	- NIU-ME - 34 eyes (26 patients)	- VA: improved in 59% - CMT: improved in 18% - ME resolution in 71% - IOP: increase of 4.4 mmHg - Cataract in 1 eye after 2.5 years	- Improved VA and CMT - Manageable IOP and cataract
Ajamil-Rodanes et al. [29]	2022, UK, Retrospective, non-comparative study	Mean 31 M (12–36 M)	- Birdshot retinochoroiditis - 15 eyes (11 patients)	- No leakage on FA in 73% at 6–12 M - ME resolution in 100% by 6 M - Persistent hypofluorescent lesions on ICGA - ERG: retinal function improved in 8/15 eyes	- Effective control of ME and vascular leakage - Limited effect on choroidal granuloma
Pavesio et al. [30]	2022, USA, Post hoc subgroup analysis of an RCT	36 M	- Bilateral NIU - 118 eyes (59 patients) - FAI study eye vs. fellow eye (conventional treatment)	- VA: gain of +9.6 letters in FAI eyes compared to loss of 4.4 letters in fellow eyes - More recurrence-free in FAI eyes than fellow eyes (29% vs. 5%) - Less topical corticosteroids in FAI eyes - Less local injections in FAI eyes - No difference in IOP-lowering surgery - More cataract surgery in phakic FAI eyes	- Effective control of inflammation - Improved VA - Less adjunctive therapy
Battista et al. [18]	2022, Italy, Retrospective, non-comparative study	12 M	- NIU-ME - 10 eyes (7 patients)	- VA: improved from 0.67 to 0.45 logMAR - CMT: decreased from 449 to 336 μm - Choroidal thickness: decreased from 251 to 190 μm - No adverse events	- Improved VA, CMT - Manageable IOP
Studsgaard et al. [17]	2022, Denmark, Retrospective, non-comparative study	2.3 ± 1.1 years (range not available)	- NIU-ME - 20 eyes (20 patients)	- VA: improved at 18 and 24 M - CMT: decreased by 48 μm at 24 M - Systemic corticosteroid discontinued in 3/5 patients - Systemic DMARD discontinued in 5/10 patients - No new-onset glaucoma, but surgery performed in 2/8 patients	- Effective control of inflammation - Improved VA - Less adjunctive therapy
Biswas et al. [23]	2023, India, Randomized controlled trial	36 M	- NIU - 153 eyes (153 patients): FAI (101 eyes) vs. sham (52 eyes)	- No significant difference in the number of recurrences and visual gain - Less recurrence at 6 M, 12 M, 36 M in FAI eyes - Less local adjuvant therapy in FAI eyes - No significant difference in IOP-related variables - More cataracts in FAI eyes	- Effective control of inflammation - No difference in visual outcome - Manageable IOP
Buhl et al. [26]	2023, Germany, Retrospective, non-comparative study	12 M	- NIU - 76 eyes (57 patients)	- VA: stable (63.21 to 62.95 letters) - CMT: decreased from 363 to 309 μm - Inflammation: reduced SUN score until 9 M (0.82 to 0.3) - No significant difference in IOP - Cataracts in 20% of phakic eyes	- Effective control of inflammation
Kessler et al. [16]	2023, Germany, Retrospective, non-comparative study	24 M	- NIU-ME - 23 eyes (23 patients)	- VA: improved from 0.50 to 0.36 logMAR (AUC: 0.41 logMAR per 6M) - CMT: decreased from 401 to 293 μm (AUC: 320 μm per 6M)	- Improved VA and CMT over 24 M - Baseline VA: the strongest predictor for AUC of BCVA
Moll-Udina et al. [31]	2023, Spain, Prospective, non-comparative study	12 M	- NIU-ME - 26 eyes (22 patients)	- VA, CMT: improved in 68% of eyes at all time points - Anterior chamber cells of ≥0.5+: decreased from 27% to 8% of eyes - Vitreous haze of ≥0.5+: decreased from 23% to 0% of eyes - Decreased use of corticosteroid and immunomodulatory therapy - No significant difference in IOP	- Effective control of inflammation
Kriegel et al. [32]	2023, Germany, Retrospective, comparative study	3 M	- NIU - 23 eyes (18 patients): DEX vs. subsequent FAI in same eyes	- VA: improved with both implants - CMT: decreased with both implants, more reduction with DEX - IOP: significant increase with DEX (not with FAI)	- Improved VA and CMT - Manageable IOP (FAI-treated eyes)
Pockar et al. [15]	2023, UK, Retrospective, non-comparative study	12 M	- NIU-ME - 11 eyes (9 patients)	- VA: stable - CRT: decreased from 435 to 296 μm - No recurrence of inflammation - Adjunctive therapy in 2 eyes - IOP-lowering drops needed in 4 eyes	- Effective control of inflammation - Improved CMT - Manageable IOP
Jabbour et al. [14]	2024, France, Retrospective, non-comparative, multi-center study	Mean 11 M (3–12 M)	- NIU-ME - 26 eyes (22 patients)	- VA, CMT: improved at 1, 3, 6, and 12 M - No rescue therapy with DEX - IOP-lowering drops needed in 19%	- Improved VA and CMT - -Manageable IOP
Abu Arif et al. [33]	2024, Germany, Retrospective, comparative study	36 M	- NIU - 135 eyes (135 patients): Responders vs. non-responders	- VA: improved at 6 M and remained stable in responders/aggravated in non-responders - CMT: decreased from 369 to 253 μm in responders/no change in non-responders - IOP-lowering therapy needed in 43% by 6 M	- Improved VA and CMT in responders - Older age and more DEX noted in non-responders
Kozak et al. [34]	2024, Middle East, Retrospective, non-comparative study	Mean 30 M (3–54 M)	- NIU - 18 eyes (13 patients)	- VA: improved from 58 to 68 letters - CMT: decreased from 407 to 326 μm - Anterior chamber cells: improved by 6 M - Vitritis score: improved by 6 M - No significant difference in IOP	- Improved VA and CMT - Stable IOP

AUC: area under the curve; CMT: central macular thickness; DEX: dexamethasone implant; FA, fluorescein angiography; FAI: fluocinolone acetonide implant; ICGA: indocyanine green angiography; IOP: intraocular pressure; M: months; ME: macular edema; NIU: non-infectious uveitis; RCT: randomized controlled study; VA: visual acuity; VKH: Vogt–Koyanagi–Harada disease.

## Data Availability

The original contributions presented in this study are included in the article/supplementary material. Further inquiries can be directed to the corresponding author.

## Supplementary Materials

The following supporting information can be downloaded at: https://www.mdpi.com/article/10.3390/biomedicines13020248/s1, File S1: Details of the literature search in the PubMed, EMBASE, and Cochrane Library databases; Table S1: Quality assessment of controlled intervention studies.
