# Supplementary material for: A Meta-Analysis of the Efficacy and Safety of the 0.19 mg Fluocinolone Acetonide Implant in Non-Infectious Uveitis
Source: Biomedicines. 2025 Jan 21;13(2):248. doi: 10.3390/biomedicines13020248 (PMC11852598; doi:10.3390/biomedicines13020248)
Supplement: Supplementary file 1 [file biomedicines-13-00248-s001.zip › File S1_query.pdf]

PubMed

No.	Query	Results
#9	#8 NOT ("Case Reports"[Publication Type] OR "case report*"[ti])	**361**
#8	#4 OR #5 OR #6 OR #7	423
#7	#2 AND #3	141
#6	#2 AND #1	411
#5	#2 AND ("versus"[ti] OR "vs"[ti] OR "compar*"[ti] OR "between"[ti] OR "than"[ti] OR "systemic"[ti] OR "standard"[ti] OR "other"[ti])	35
#4	"fluocinolone acetonide implant versus"[tiab:~3] OR "fluocinolone acetonide implants versus"[tiab:~3] OR "fluocinolone acetonide implantation versus"[tiab:~3] OR "fluocinolone implant versus"[tiab:~3] OR "fluocinolone implants versus"[tiab:~3] OR "fluocinolone implantation versus"[tiab:~3] OR "fluocinolone acetonide insert versus"[tiab:~3] OR "fluocinolone acetonide inserts versus"[tiab:~3] OR "fluocinolone acetonide insertion versus"[tiab:~3] OR "fluocinolone insert versus"[tiab:~3] OR "fluocinolone inserts versus"[tiab:~3] OR "fluocinolone insertion versus"[tiab:~3] OR "intravitreal fluocinolone versus"[tiab:~3] OR "intra-vitreal fluocinolone versus"[tiab:~3] OR "flucinolone acetonide implant versus"[tiab:~3] OR "flucinolone acetonide implants versus"[tiab:~3] OR "flucinolone acetonide implantation versus"[tiab:~3] OR "flucinolone implant versus"[tiab:~3] OR "flucinolone implants versus"[tiab:~3] OR "flucinolone implantation versus"[tiab:~3] OR "flucinolone acetonide insert versus"[tiab:~3] OR "flucinolone acetonide inserts versus"[tiab:~3] OR "flucinolone acetonide insertion versus"[tiab:~3] OR "flucinolone insert versus"[tiab:~3] OR "flucinolone inserts versus"[tiab:~3] OR "flucinolone insertion versus"[tiab:~3] OR "intravitreal flucinolone versus"[tiab:~3] OR "intra-vitreal flucinolone versus"[tiab:~3] OR "FAc implant versus"[tiab:~3] OR "FAc implants versus"[tiab:~3] OR "FAc implantation versus"[tiab:~3] OR "FAc insert versus"[tiab:~3] OR "FAc inserts versus"[tiab:~3] OR "FAc insertion versus"[tiab:~3] OR "FA implant versus"[tiab:~3] OR "FA implants versus"[tiab:~3] OR "FA implantation versus"[tiab:~3] OR "FA insert versus"[tiab:~3] OR "FA inserts versus"[tiab:~3] OR "FA insertion versus"[tiab:~3] OR "fluocinolone acetonide implant vs"[tiab:~3] OR "fluocinolone acetonide implants vs"[tiab:~3] OR "fluocinolone acetonide implantation vs"[tiab:~3] OR "fluocinolone implant vs"[tiab:~3] OR "fluocinolone implants vs"[tiab:~3] OR "fluocinolone implantation vs"[tiab:~3] OR "fluocinolone acetonide insert vs"[tiab:~3] OR "fluocinolone acetonide inserts vs"[tiab:~3] OR "fluocinolone acetonide insertion vs"[tiab:~3] OR "fluocinolone insert vs"[tiab:~3] OR "fluocinolone inserts vs"[tiab:~3] OR "fluocinolone insertion vs"[tiab:~3] OR "intravitreal fluocinolone vs"[tiab:~3] OR "intra-vitreal fluocinolone vs"[tiab:~3] OR "flucinolone acetonide implant vs"[tiab:~3] OR "flucinolone acetonide implants vs"[tiab:~3] OR "flucinolone acetonide implantation vs"[tiab:~3] OR "flucinolone implant vs"[tiab:~3] OR "flucinolone implants vs"[tiab:~3] OR "flucinolone implantation vs"[tiab:~3] OR "flucinolone acetonide insert vs"[tiab:~3] OR "flucinolone acetonide inserts vs"[tiab:~3] OR "flucinolone acetonide insertion vs"[tiab:~3] OR "flucinolone insert vs"[tiab:~3] OR "flucinolone inserts vs"[tiab:~3] OR "flucinolone insertion vs"[tiab:~3] OR "intravitreal flucinolone vs"[tiab:~3] OR "intra-vitreal flucinolone vs"[tiab:~3] OR "FAc implant vs"[tiab:~3] OR "FAc implants vs"[tiab:~3] OR "FAc implantation vs"[tiab:~3] OR "FAc insert vs"[tiab:~3] OR "FAc inserts vs"[tiab:~3] OR "FAc insertion vs"[tiab:~3] OR "FA implant vs"[tiab:~3] OR "FA implants vs"[tiab:~3] OR "FA implantation vs"[tiab:~3] OR "FA insert vs"[tiab:~3] OR "FA inserts vs"[tiab:~3] OR "FA insertion vs"[tiab:~3] OR "fluocinolone acetonide implant comparisons"[tiab:~3] OR "fluocinolone acetonide implants comparisons"[tiab:~3] OR "fluocinolone acetonide implantation comparisons"[tiab:~3] OR "fluocinolone implant comparisons"[tiab:~3] OR "fluocinolone implants comparisons"[tiab:~3] OR "fluocinolone implantation comparisons"[tiab:~3] OR "fluocinolone acetonide insert comparisons"[tiab:~3] OR "fluocinolone acetonide inserts comparisons"[tiab:~3] OR "fluocinolone acetonide insertion comparisons"[tiab:~3] OR "fluocinolone insert comparisons"[tiab:~3] OR "fluocinolone inserts comparisons"[tiab:~3] OR "fluocinolone insertion comparisons"[tiab:~3] OR "intravitreal fluocinolone comparisons"[tiab:~3] OR "intra-vitreal fluocinolone comparisons"[tiab:~3] OR "flucinolone acetonide implant comparisons"[tiab:~3] OR "flucinolone acetonide implants comparisons"[tiab:~3] OR "flucinolone acetonide implantation comparisons"[tiab:~3] OR "flucinolone implant comparisons"[tiab:~3] OR "flucinolone implants comparisons"[tiab:~3] OR "flucinolone implantation comparisons"[tiab:~3] OR "flucinolone acetonide insert comparisons"[tiab:~3] OR "flucinolone acetonide inserts comparisons"[tiab:~3] OR "flucinolone acetonide insertion comparisons"[tiab:~3] OR "flucinolone insert comparisons"[tiab:~3] OR "flucinolone inserts comparisons"[tiab:~3] OR "flucinolone insertion comparisons"[tiab:~3] OR "intravitreal flucinolone comparisons"[tiab:~3] OR "intra-vitreal flucinolone comparisons"[tiab:~3] OR "FAc implant comparisons"[tiab:~3] OR "FAc implants comparisons"[tiab:~3] OR "FAc implantation	64
#3	"Triamcinolone"[mesh] OR "Dexamethasone"[mesh] OR "sham"[tiab] OR "triamcinolone"[tiab] OR "volon"[tiab] OR "aristocort"[tiab] OR "dexamethasone"[tiab] OR "DEX"[tiab] OR "hexadecadrol"[tiab] OR "decaspray"[tiab] OR "dexasone"[tiab] OR "dexpak"[tiab] OR "maxidex"[tiab] OR "millicorten"[tiab] OR "oradexon"[tiab] OR "decaject"[tiab] OR "hexadrol"[tiab]	200,344

#2	("Fluocinolone Acetonide"[mesh] AND "Drug Implants"[mesh]) OR "fluocinolone acetonide implant"[tiab:~3] OR "fluocinolone acetonide implants"[tiab:~3] OR "fluocinolone acetonide implantation"[tiab:~3] OR "intravitreal fluocinolone acetonide"[tiab:~3] OR "intra-vitreal fluocinolone acetonide"[tiab:~3] OR "fluocinolone implant"[tiab:~3] OR "fluocinolone implants"[tiab:~3] OR "fluocinolone implantation"[tiab:~3] OR "fluocinolone insert"[tiab:~3] OR "fluocinolone inserts"[tiab:~3] OR "fluocinolone insertion"[tiab:~3] OR "fluocinolone intravitreal"[tiab:~3] OR "fluocinolone intra-vitreal"[tiab:~3] OR "flucinolone acetonide implant"[tiab:~3] OR "flucinolone acetonide implants"[tiab:~3] OR "flucinolone acetonide implantation"[tiab:~3] OR "intravitreal flucinolone acetonide"[tiab:~3] OR "intra-vitreal flucinolone acetonide"[tiab:~3] OR "flucinolone implant"[tiab:~3] OR "flucinolone implants"[tiab:~3] OR "flucinolone implantation"[tiab:~3] OR "flucinolone insert"[tiab:~3] OR "flucinolone inserts"[tiab:~3] OR "flucinolone insertion"[tiab:~3] OR "flucinolone intravitreal"[tiab:~3] OR "flucinolone intra-vitreal"[tiab:~3] OR "FAc implant"[tiab:~3] OR "FAc implants"[tiab:~3] OR "FAc implantation"[tiab:~3] OR "FA implant"[tiab:~3] OR "FA implants"[tiab:~3] OR "FA implantion"[tiab:~3] OR (("fluocinolone"[ti] OR "flucinolone"[ti]) AND ("acetonide"[ti] OR "FAc"[ti] OR "FA"[ti]) AND ("implant*"[tiab] OR "intravitreal"[tiab] OR "intra-vitreal"[tiab] OR "insert*"[tiab] OR "device*"[tiab] OR "pellet*"[tiab])) OR "yutiq"[tiab] OR "iluvien"[tiab] OR "illuvien"[tiab] OR "retisert"[tiab]	463
#1	"Uveitis"[mesh] OR "Macular Edema"[mesh] OR "Glaucoma"[mesh] OR "Cataract"[mesh] OR "Intraocular Pressure"[mesh] OR "Visual Acuity"[mesh] OR "Vitreous Body"[mesh] OR "uveitis"[tiab] OR "panuveitis"[tiab] OR "uveitides"[tiab] OR "macula edema"[tiab] OR "macula oedema"[tiab] OR "macula lutea edema"[tiab] OR "macular edema"[tiab] OR "macular oedema"[tiab] OR "glaucoma"[tiab] OR "cataract"[tiab] OR "cataractous lens"[tiab] OR "eye lens opacity"[tiab] OR "lens clouding"[tiab] OR "lens opacity"[tiab] OR "posterior capsule opacification"[tiab] OR "retina thickness"[tiab:~3] OR "retinal thickness"[tiab:~3] OR "macula thickness"[tiab:~3] OR "macular thickness"[tiab:~3] OR "fovea thickness"[tiab:~3] OR "foveal thickness"[tiab:~3] OR "intraocular pressure"[tiab] OR "eye ball pressure"[tiab] OR "eye pressure"[tiab] OR "eye tension"[tiab] OR "eyeball pressure"[tiab] OR "intraocular tension"[tiab] OR "intraorbital pressure"[tiab] OR "ocular pressure"[tiab] OR "ocular tension"[tiab] OR "visual acuity"[tiab] OR "visual resolution"[tiab] OR "visual sharpness"[tiab] OR "vitreous"[tiab] OR "camera vitrea bulbi"[tiab] OR "corpus vitreum"[tiab] OR "humor vitreus"[tiab] OR "vitreum"[tiab]	326,943

Embase

No.	Query	Results
#9	#8 NOT ('case report'/de OR 'case report*':ti,kw)	**802**
#8	#4 OR #5 OR #6 OR #7	916
#7	#2 AND #3	539
#6	#2 AND #1	893
#5	#2 AND ('versus':ti OR 'vs':ti OR 'compar*':ti OR 'between':ti OR 'than':ti OR 'systemic':ti OR 'standard':ti OR 'other':ti)	63
#4	(('fluocinolone acetonide implant*' OR 'fluocinolone implant*' OR 'fluocinolone acetonide insert*' OR 'fluocinolone insert*' OR 'intravitreal fluocinolone' OR 'intra-vitreal fluocinolone' OR 'flucinolone acetonide implant*' OR 'flucinolone implant*' OR 'flucinolone acetonide insert*' OR 'flucinolone insert*' OR 'intravitreal flucinolone' OR 'intra-vitreal flucinolone' OR 'fac implant*' OR 'fac insert*' OR 'fa implant*' OR 'fa insert*') NEAR/3 ('versus' OR 'vs' OR 'compar*' OR 'between' OR 'than' OR 'systemic' OR 'standard' OR 'other')):ti,ab,kw	63
#3	('triamcinolone'/exp OR 'dexamethasone'/exp OR 'sham':ti,ab,kw OR 'triamcinolone':ti,ab,kw OR 'volon':ti,ab,kw OR 'aristocort':ti,ab,kw OR 'dexamethasone':ti,ab,kw OR 'dex':ti,ab,kw OR 'hexadecadrol':ti,ab,kw OR 'decaspray':ti,ab,kw OR 'dexasone':ti,ab,kw OR 'dexpak':ti,ab,kw OR 'maxidex':ti,ab,kw OR 'millicorten':ti,ab,kw OR 'oradexon':ti,ab,kw OR 'decaject':ti,ab,kw OR 'hexadrol':ti,ab,kw)	383,546

#2	('fluocinolone acetonide'/exp AND ('ophthalmological implant'/exp OR 'intravitreal implant'/exp OR 'implant'/exp OR 'drug implant'/exp OR 'ophthalmic drug delivery device'/exp) OR (('fluocinolone' NEAR/3 'acetonide' NEAR/3 'implant*'):ti,ab,kw) OR (('intravitreal' NEAR/3 'fluocinolone' NEAR/3 'acetonide'):ti,ab,kw) OR (('intra-vitreal' NEAR/3 'fluocinolone' NEAR/3 'acetonide'):ti,ab,kw) OR (('fluocinolone' NEAR/3 'implant*'):ti,ab,kw) OR (('fluocinolone' NEAR/3 'insert*'):ti,ab,kw) OR (('fluocinolone' NEAR/3 'intravitreal*'):ti,ab,kw) OR (('fluocinolone' NEAR/3 'intra-vitreal*'):ti,ab,kw) OR (('flucinolone' NEAR/3 'acetonide' NEAR/3 'implant*'):ti,ab,kw) OR (('intravitreal' NEAR/3 'flucinolone' NEAR/3 'acetonide'):ti,ab,kw) OR (('intra-vitreal' NEAR/3 'flucinolone' NEAR/3 'acetonide'):ti,ab,kw) OR (('flucinolone' NEAR/3 'implant*'):ti,ab,kw) OR (('flucinolone' NEAR/3 'insert*'):ti,ab,kw) OR (('flucinolone' NEAR/3 'intravitreal*'):ti,ab,kw) OR (('flucinolone' NEAR/3 'intra-vitreal*'):ti,ab,kw) OR (('fac' NEAR/3 'implant*'):ti,ab,kw) OR (('fa' NEAR/3 'implant*'):ti,ab,kw) OR (('fluocinolone':ti,kw OR 'flucinolone':ti,kw) AND ('acetonide':ti,kw OR 'fac':ti,kw OR 'fa':ti,kw) AND ('implant*':ti,ab,kw OR 'intravitreal':ti,ab,kw OR 'intra-vitreal':ti,ab,kw OR 'insert*':ti,ab,kw OR 'device*':ti,ab,kw OR 'pellet*':ti,ab,kw)) OR 'yutiq':ti,ab,kw OR 'iluvien':ti,ab,kw OR 'illuvien':ti,ab,kw OR 'retisert':ti,ab,kw)	992
#1	('uveitis'/exp OR 'macular edema'/exp OR 'glaucoma'/exp OR 'cataract'/exp OR 'retinal thickness'/exp OR 'intraocular pressure'/exp OR 'visual acuity'/exp OR 'vitreous body'/exp OR 'uveitis':ti,ab,kw OR 'panuveitis':ti,ab,kw OR 'uveitides':ti,ab,kw OR 'macula edema':ti,ab,kw OR 'macula oedema':ti,ab,kw OR 'macula lutea edema':ti,ab,kw OR 'macular edema':ti,ab,kw OR 'macular oedema':ti,ab,kw OR 'glaucoma':ti,ab,kw OR 'cataract':ti,ab,kw OR 'cataractous lens':ti,ab,kw OR 'eye lens opacity':ti,ab,kw OR 'lens clouding':ti,ab,kw OR 'lens opacity':ti,ab,kw OR 'posterior capsule opacification':ti,ab,kw OR ((('retina*' OR 'macula*' OR 'fovea*') NEAR/3 'thickness'):ti,ab,kw) OR 'intraocular pressure':ti,ab,kw OR 'eye ball pressure':ti,ab,kw OR 'eye pressure':ti,ab,kw OR 'eye tension':ti,ab,kw OR 'eyeball pressure':ti,ab,kw OR 'intraocular tension':ti,ab,kw OR 'intraorbital pressure':ti,ab,kw OR 'ocular pressure':ti,ab,kw OR 'ocular tension':ti,ab,kw OR 'visual acuity':ti,ab,kw OR 'visual resolution':ti,ab,kw OR 'visual sharpness':ti,ab,kw OR 'vitreous':ti,ab,kw OR 'camera vitrea bulbi':ti,ab,kw OR 'corpus vitreum':ti,ab,kw OR 'humor vitreus':ti,ab,kw OR 'vitreum':ti,ab,kw)	464,511

Cochrane CENTRAL

No.	Query	Results
#9	#8 NOT case-report*:ti,kw in Trials	**148**
#8	#4 OR #5 OR #6 OR #7	150
#7	#2 AND #3	49
#6	#2 AND #1	148
#5	#2 AND (versus:ti OR vs:ti OR compar*:ti OR between:ti OR than:ti OR systemic:ti OR standard:ti OR other:ti)	33
#4	((fluocinolone-acetonide-implant* OR fluocinolone-implant* OR fluocinolone-acetonide-insert* OR fluocinolone-insert* OR intravitreal-fluocinolone OR intra-vitreal-fluocinolone OR flucinolone-acetonide-implant* OR flucinolone-implant* OR flucinolone-acetonide-insert* OR flucinolone-insert* OR intravitreal-flucinolone OR intra-vitreal-flucinolone OR FAc-implant* OR FAc-insert* OR FA-implant* OR FA-insert*) NEAR/3 (versus OR vs OR compar* OR between OR than OR systemic OR standard OR other)):ti,ab,kw	37
#3	([mh "Triamcinolone"] OR [mh "Dexamethasone"] OR sham:ti,ab,kw OR triamcinolone:ti,ab,kw OR volon:ti,ab,kw OR aristocort:ti,ab,kw OR dexamethasone:ti,ab,kw OR DEX:ti,ab,kw OR hexadecadrol:ti,ab,kw OR decaspray:ti,ab,kw OR dexasone:ti,ab,kw OR dexpak:ti,ab,kw OR maxidex:ti,ab,kw OR millicorten:ti,ab,kw OR oradexon:ti,ab,kw OR decaject:ti,ab,kw OR hexadrol:ti,ab,kw)	47,548

#2	([mh "Fluocinolone Acetonide"] AND [mh "Drug Implants"]) OR (fluocinolone NEAR/3 acetonide NEAR/3 implant*):ti,ab,kw OR (intravitreal NEAR/3 fluocinolone NEAR/3 acetonide):ti,ab,kw OR (intra-vitreal NEAR/3 fluocinolone NEAR/3 acetonide):ti,ab,kw OR (fluocinolone NEAR/3 implant*):ti,ab,kw OR (fluocinolone NEAR/3 insert*):ti,ab,kw OR (fluocinolone NEAR/3 intravitreal*):ti,ab,kw OR (fluocinolone NEAR/3 intra-vitreal*):ti,ab,kw OR (flucinolone NEAR/3 acetonide NEAR/3 implant*):ti,ab,kw OR (intravitreal NEAR/3 flucinolone NEAR/3 acetonide):ti,ab,kw OR (intra-vitreal NEAR/3 flucinolone NEAR/3 acetonide):ti,ab,kw OR (flucinolone NEAR/3 implant*):ti,ab,kw OR (flucinolone NEAR/3 insert*):ti,ab,kw OR (flucinolone NEAR/3 intravitreal*):ti,ab,kw OR (flucinolone NEAR/3 intra-vitreal*):ti,ab,kw OR (FAc NEAR/3 implant*):ti,ab,kw OR (FA NEAR/3 implant*):ti,ab,kw OR ((fluocinolone:ti,kw OR flucinolone:ti,kw) AND (acetonide:ti,kw OR FAc:ti,kw OR FA:ti,kw) AND (implant*:ti,ab,kw OR intravitreal:ti,ab,kw OR intra-vitreal:ti,ab,kw OR insert*:ti,ab,kw OR device*:ti,ab,kw OR pellet*:ti,ab,kw)) OR yutiq:ti,ab,kw OR iluvien:ti,ab,kw OR illuvien:ti,ab,kw OR retisert:ti,ab,kw	156
#1	[mh "Uveitis"] OR [mh "Macular Edema"] OR [mh "Glaucoma"] OR [mh "Cataract"] OR [mh "Intraocular Pressure"] OR [mh "Visual Acuity"] OR [mh "Vitreous Body"] OR uveitis:ti,ab,kw OR panuveitis:ti,ab,kw OR uveitides:ti,ab,kw OR macula-edema:ti,ab,kw OR macula-oedema:ti,ab,kw OR macula-lutea-edema:ti,ab,kw OR macular-edema:ti,ab,kw OR macular-oedema:ti,ab,kw OR glaucoma:ti,ab,kw OR cataract:ti,ab,kw OR cataractous-lens:ti,ab,kw OR eye-lens-opacity:ti,ab,kw OR lens-clouding:ti,ab,kw OR lens-opacity:ti,ab,kw OR posterior-capsule-opacification:ti,ab,kw OR ((retina* OR macula* OR fovea*) NEAR/3 thickness):ti,ab,kw OR intraocular-pressure:ti,ab,kw OR eye-ball-pressure:ti,ab,kw OR eye-pressure:ti,ab,kw OR eye-tension:ti,ab,kw OR eyeball-pressure:ti,ab,kw OR intraocular-tension:ti,ab,kw OR intraorbital-pressure:ti,ab,kw OR ocular-pressure:ti,ab,kw OR ocular-tension:ti,ab,kw OR visual-acuity:ti,ab,kw OR visual-resolution:ti,ab,kw OR visual-sharpness:ti,ab,kw OR vitreous:ti,ab,kw OR camera-vitrea-bulbi:ti,ab,kw OR corpus-vitreum:ti,ab,kw OR humor-vitreus:ti,ab,kw OR vitreum:ti,ab,kw	34,523
